# Epoxide Hydrolase Inhibitors for the Treatment of Alzheimer’s Disease and Other Neurological Disorders: A Comprehensive Review

**DOI:** 10.3390/biomedicines13092073

**Published:** 2025-08-26

**Authors:** Manal Abdalla, Mohamed Ibrahim, Noora Alkorbi, Shaika Alkuwari, Shona Pedersen, Hassaan Anwer Rathore

**Affiliations:** 1 Department of Basic Medical Science, College of Medicine, QU Health, Qatar University, Doha 2713, Qatar; ma2004240@qu.edu.qa (M.A.); mi2002561@qu.edu.qa (M.I.); na1901774@qu.edu.qa (N.A.); sa1909081@qu.edu.qa (S.A.); spedersen@qu.edu.qa (S.P.); 2 Department of Pharmaceutical Sciences, College of Pharmacy, QU Health, Qatar University, Doha 2713, Qatar

**Keywords:** Alzheimer’s disease, soluble epoxide hydrolase inhibitors, neuroinflammation, cognitive decline

## Abstract

Alzheimer’s disease is the most common form of dementia, yet current treatments only offer symptomatic relief, with little preventative, therapeutic, or disease-modifying properties. As a result, there has been growing interest in targeting various disease mechanisms. One promising target is soluble epoxide hydrolase (sEH), an enzyme found in many organs, playing an important role in metabolism and detoxification. In the brain, sEH is mainly present in astrocytes, oligodendrocytes, and neuronal cell bodies, with higher concentrations in the cerebral cortex and striatum. The main function of sEH is the hydrolysis of epoxyeicosatrienoic acids (EETs), which are important anti-inflammatory molecules derived from arachidonic acid. Deletion of EPHX2, the encoding gene of sEH, maintains EET levels in the brain and helps mitigate inflammation. Multiple studies have found links between sEH function, inflammation, and neurodegeneration in Alzheimer’s disease. Several compounds, including TPPU, benzohomoadamantane derivatives, and natural products, have shown significant beneficial effects, including reduction of amyloid-beta plaques, tau fibrils, and inflammation, while improving cognition and neuronal structure and function. sEH inhibitors have also been explored for their potential in the management of Parkinson’s disease, vascular dementia, stroke, and other neurodegenerative conditions. Although these preclinical findings are promising, efficacy and safety concerns still need to be addressed, and further clinical trials are needed to translate these therapeutic agents into clinical practice.

## 1. Introduction

As our societies continue to evolve and the field of medicine advances significantly, life expectancies have increased substantially. This demographic shift is associated with a rising prevalence of cognitive impairment [1]. This condition typically progresses through a preclinical stage, followed by mild cognitive impairment, and ultimately, dementia. Currently, about 57 million people are living with dementia, including its various subtypes, such as Alzheimer’s disease, vascular dementia, frontotemporal dementia, Lewy body dementia, and others. This number is expected to rise to 78 million by 2030 [2].

The most prevalent subtype of dementia, accounting for 60–70% of dementia cases, is Alzheimer’s disease (AD) [2]. This condition not only affects patients but also places a significant burden on families, societies, and healthcare systems [3]. Multiple hypotheses have been proposed to explain the pathogenesis of Alzheimer’s disease (AD). The most widely discussed mechanisms include amyloid-beta (Aβ) plaque accumulation, tau protein hyperphosphorylation, and the resulting neuroinflammation and degeneration. Aβ plaques are insoluble, neurotoxic peptides formed due to aberrant processing of amyloid precursor protein (APP) by beta- and gamma-secretases instead of alpha-secretase. The excessive phosphorylation of tau protein, which normally stabilizes microtubules to preserve neuronal structure and function, results in the creation of aggregates and neurofibrillary tangles. These two processes stimulate microglial cells and astrocytes to secrete proinflammatory cytokines such as interleukin-1β and tumor necrosis factor-alpha (TNF-α), which subsequently cause synaptic degeneration and neurotransmitter imbalances, particularly involving acetylcholine and glutamate, along with neuronal loss, especially in memory-associated regions of the brain like the hippocampus and entorhinal cortex. This cascade contributes to cognitive decline. Furthermore, several genes have been associated with Alzheimer’s disease. The most extensively investigated include presenilin 1 (PSEN1), presenilin 2 (PSEN2), and amyloid precursor protein (APP) mutations, all of which contribute to elevated Aβ deposition and have been linked to familial Alzheimer’s disease. In addition, the epsilon 4 (ε4) variant of the apolipoprotein E (APOE) gene is a well-established genetic risk factor for sporadic Alzheimer’s disease, as it has been shown to reduce Aβ clearance by astrocytes and amplify tau phosphorylation, thereby exacerbating downstream neuroinflammatory effects [4,5,6].

The current treatment regimen for Alzheimer’s disease includes multiple pharmacological agents. Cholinesterase inhibitors, mainly donepezil, rivastigmine, and galantamine, are mainly used in mild to moderate disease conditions [7]. Memantine, an inhibitor of the N-methyl-D-aspartate (NMDA) receptor, is used for moderate to severe Alzheimer’s disease [8]. Additionally, two anti-amyloid antibodies, namely Lecanemab and Donanemab [9], have recently been approved for use in early mild Alzheimer’s disease.

Some of the challenges associated with current treatment regimens include the lack of effective therapeutic and preventive agents, as most available medications are primarily used for symptomatic management. In addition, difficulties in delivering drugs to the central nervous system (CNS), along with the complex disease mechanisms involving multiple pathways and genetic, epigenetic, and environmental factors, further complicate treatment. Therefore, the role of precision pharmacology becomes increasingly important. Many agents are currently under study, including tau protein dephosphorylators and degraders, such as proteolysis-targeting chimeras (PROTACs) [4,10]. Other techniques include covalent and allosteric modulation of several molecules involved in Alzheimer’s disease (AD) pathogenesis, such as gamma-secretase and glycogen synthase kinase 3 beta (GSK-3β). Also, enzymes like monoamine oxidase B (MAO-B), phosphodiesterase (PDE), butyrylcholinesterase (BuChE), and beta secretase are targets of therapeutic interest. Even vaccines that prevent amyloid plaques and probiotics that affect the gut–brain axis, which has been linked to the disease, have shown potential in Alzheimer’s disease treatment. Although these agents are promising, they are still under investigation for safety and efficacy for use in humans [4,10,11].

One such molecule that has recently emerged is the enzyme epoxide hydrolase (EH). In addition to hydrolyzing epoxy-fatty acids (EpFAs), Epoxide hydrolase functions as a phosphatase. It is expressed in multiple organ systems, and it plays a role in the metabolism and breakdown of the anti-inflammatory molecules that are generated from arachidonic acid (ARA), such as epoxyeicosatrienoic acids (EETs) and others. It has been proposed that inhibiting epoxide hydrolase has potential for the management of a spectrum of conditions across body systems [12].

Recent research studies revealed that epoxide hydrolase levels are elevated in both human and murine models of Alzheimer’s disease, indicating possible connections with the disease. Also, it was found that epoxide hydrolase inhibitors improve cognitive function in mice by reducing oxidative stress and inflammation [13].

Beyond Alzheimer’s disease, epoxide hydrolase inhibitors exhibit promising effects in treating Parkinson’s disease, depression, schizophrenia, and stroke, as well as other neurological, cardiovascular, and metabolic conditions [12].

This article presents a comprehensive review of epoxide hydrolase and its inhibitors, including the mechanisms, indications, rationale, and future recommendations for its use. The objective of this review is to consolidate the present understanding regarding epoxide hydrolase inhibitors, assess their potential for treating Alzheimer’s disease, and pinpoint prospective areas for future research.

## 2. Methodology

This review explores the current evidence on soluble epoxide hydrolase (sEH) and its inhibitors in Alzheimer’s disease and other neurological disorders. The search strategy included a combination of the terms “soluble epoxide hydrolase,” “sEH inhibitors,” “epoxyeicosatrienoic acids,” “Alzheimer’s disease,” and “neurological disorders.” Relevant articles were retrieved from PubMed, Google Scholar, and Scopus, and included original in vivo and ex vivo experiments, clinical research, preclinical investigations, mechanistic analyses, pharmacological studies, and review articles published in English. The search was performed from database inception up to August 2025. Studies were selected based on their relevance to the mechanisms, therapeutic potential, and neuroprotective effects of sEH inhibitors in Alzheimer’s disease and related neurological conditions. The search included Alzheimer’s disease; soluble epoxide hydrolase inhibitors; neuroinflammation; and cognitive decline as key words. Studies that were not written in English or investigated sEH in diseased conditions other than neurological deficit diseases were excluded. Studies were analyzed thematically according to key features of sEH inhibitors, including their biochemical mechanisms, therapeutic applications, and roles in Alzheimer’s disease and other CNS disorders. Emphasis was placed on selecting recent, high-quality evidence to provide a comprehensive overview and identify future directions in the field.

## 3. Epoxide Hydrolase

### 3.1. The Enzyme

Epoxide hydrolases (EHs) catalyze the hydrolysis of epoxides to their corresponding diols in a cofactor-independent manner [14]. These enzymes play a crucial role in the biotransformation of endogenous epoxides and the detoxification of xenobiotic compounds. Being widely distributed across both prokaryotic and eukaryotic organisms, EHs in humans are classified within the α/β-hydrolase fold family, with a systematic nomenclature distinguishing the different isoforms: EPHX1 encodes microsomal epoxide hydrolase (mEH), while EPHX2 encodes soluble epoxide hydrolase (sEH). Among these, sEH is the most important isoform in the hydrolysis of EpFAs, followed by mEH [15].

sEH is the key enzyme involved in the metabolism of eicosanoid epoxides. It is composed of two monomers, each containing a C-terminal hydrolase domain, which functions through a two-step mechanism involving a nucleophilic attack by aspartic acid followed by hydrolysis, and an N-terminal phosphatase domain, which hydrolyzes lipid phosphates [16].

Soluble epoxide hydrolase (sEH) is found in inflammatory cells and among different organs, such as liver, kidney, lungs, heart, brain, spleen, intestine, adrenals, urinary bladder, skin, placenta, mammary gland, and testis [17], as well as in vascular endothelial and smooth muscle cells [18]. At the subcellular level, sEH is mostly located in the cytosol or peroxisomes [19]. In the brain, it is widely distributed in the brain’s cerebral cortex and striatum. Primarily, it is found in astrocytes, oligodendrocytes, neuronal cell bodies, and smooth muscles of cerebral blood arteries, suggesting a role in regulating cerebral blood flow (CBF), brain activity, and pathogenesis of neurological disorders [20].

The expression of sEH can be upregulated by agonists of peroxisome proliferator-activated receptor-α (PPARα), peroxisome proliferator-activated receptor-γ (PPARγ), such as fibrates and glitazones, angiotensin II, and sex hormones [21,22]. PPARγ agonists were found to upregulate the expression of sEH in adipose tissues and downregulate it in cardiomyocytes [23]. Additionally, sEH expression increases during inflammation, making sEH a potential inflammatory marker [24]. Moreover, elevated sEH activity has been associated with a reduction in endogenous sEH inhibitors, such as specific metal cations, during systemic inflammation [25].

In contrast, microsomal epoxide hydrolase (mEH) is primarily recognized as a detoxification enzyme, catalyzing the hydrolysis of xenobiotic epoxides [26]. mEH is expressed in almost all tissues but mainly resides in the endoplasmic reticulum membrane of hepatic cells [27]. Several genetic variations of mEH have been linked to increased susceptibility to a range of diseases and cancers [28]. Notably, mEH detected in the bloodstream has been referred to as a paraneoplastic antigen and has been explored as a biomarker for liver neoplasms and other hepatic diseases [29]. In addition, a study identified mEH as a brain tumor antigen in some glioblastoma cell lines [30]. Moreover, the expression of EPHX1 has been identified in multiple brain regions, including the cerebellum, frontal and occipital lobes, pons, red nucleus, and substantia nigra. The detection of the EPHX1 protein in both neurons and astrocytes has raised the possibility of its involvement in neurotoxic processes [31]. Further evidence for its role in neurodegeneration comes from a study reporting increased EPHX1 expression in the brains of patients with Alzheimer’s disease [32]. Additionally, Animal studies have shown activity of mEH and sEH in rat brain cortical astrocytes, suggesting they may contribute to the regulation of cerebrovascular function [33]. Another study of mouse brains has also shown that EPHX1 plays a role in the cerebral metabolism of EETs, potentially influencing neuronal signaling, vascular function, and inflammation [34].

Although mEH metabolizes endogenous epoxides such as EpFAs, its activity is generally lower than that of sEH [26]. Interestingly, for certain substrates like 8,9-EET and 11,12-EET, the catalytic efficiency (kcat/KM) of mEH is comparable to that of sEH. However, mEH demonstrates markedly reduced activity toward other substrates, including 14,15-EET and 9,10-epoxyoctadecaenoic acid (9,10-EpOME). In some models, dual deletion of Ephx1 and Ephx2 genes is more effective than single knockouts in altering the epoxide/diol balance. Nonetheless, pharmacological inhibition of sEH remains the primary strategy for modulating EpFA levels [35].

Furthermore, based on protein structure homology, another two genes, EPHX3 and EPHX4, encode epoxide hydrolase 3 (EH3) and hydrolase 4 (EH4), respectively [36]. EH3 has been shown to have similar activity on EpFA as sEH. Interestingly, the genetic deletion of EH3 in mice did not produce an observable phenotype, as there were no significant changes in EpFA levels or the inflammatory response to lipopolysaccharide. This lack of effect might have been due to compensatory activity from the more abundant sEH and mEH enzymes [37]. A recent study elaborated that EH3 catalyzes the hydrolysis of linoleate-derived epoxyalcohol and ceramides, indicating a role in skin barrier function and possible contribution to other tissues [38]. In contrast, no reports on the catalytic activity of EH4 on the hydrolysis of epoxides are available [36]. EH4, encoded by the EPHX4 gene, appears to be involved in various physiological processes. For example, EPHX4 has been shown to regulate sebaceous lipid metabolism [39]. In addition, increased expression of EPHX4 has been reported in pseudomyxoma peritonei and primary rectal cancer [40,41]. More recently, EPHX4 was found to be highly expressed in laryngeal cancer specimens, suggesting its potential as an immunotherapeutic target [42].

A study [15] evaluated the activity and contribution of the recombinantly produced epoxide hydrolase isozymes (sEH, mEH, EH3, and EH4) using baculovirus vectors. Seven substrates were analyzed, utilizing radiolabeling and Liquid chromatography/tandem mass spectrometry (LC-MS/MS) techniques for substrate activity measurement. It was found that when compared to mEH, sEH demonstrated significantly higher activity on both 14,15-EET and 13,14-EDP, which was a significant two-order-of-magnitude difference in certain activities. While EH3 was more active on certain substrates (e.g., [3H]-t-DPPO and 13,14-EDP), it was not as active as sEH or mEH across the majority of substrates. EH4, on the other hand, showed no detectable activity on the tested substrates. Although mEH also had a major role, especially in the liver, adrenal glands, and lungs, especially in smokers, sEH was found to be the primary regulator of the breakdown of epoxy fatty acids in all tissues. Inhibition studies emphasized the superiority of sEH, blocking over 90% of the hydrolytic activity. However, the therapeutic effect of inhibiting sEH in some tissues may be limited by residual mEH activity.

### 3.2. Epoxyeicosatrienoic Acids (EETs) and Epoxide Hydrolase (EH) Inhibitors

Arachidonic acid (ARA), eicosapentaenoic acid (EPA), docosapentaenoic acid (DPA), and linoleic acid are all polyunsaturated fatty acids (PUFAs). The cytochrome P450 (CYP450) enzyme system metabolizes these PUFAs into various epoxy and hydroxy derivatives: it generates epoxyeicosatrienoic acids (EETs) and hydroxyeicosatetraenoic acids (HETEs) from ARA, epoxyeicosatetraenoic acids (EEQs) from EPA, epoxydocosapentenoic acids (EDPs) from DHA, and epoxyoctadecenoic acids (EpOMEs) from linoleic acid [43,44]. Collectively, these CYP450-derived metabolites are referred to as epoxy-fatty acids (EpFAs). While products of cyclooxygenase (COX) and lipoxygenase (LOX) pathways typically exhibit pro-inflammatory properties, EpFAs are known for their anti-inflammatory effects [25]. Functioning as autocrine and paracrine lipid mediators, EpFAs are rapidly hydrolyzed by epoxide hydrolases (EHs), primarily soluble epoxide hydrolase (sEH), into their corresponding diols [45]. Focusing on ARA-derived EpFAs, EETs exist as four regioisomers: 14,15-EET, 11,12-EET, 8,9-EET, and 5,6-EET, with 14,15-EET being the most abundant and exhibiting the highest affinity for sEH [46]. EETs are hydrolyzed by EHs into their less biologically active dihydroxyeicosatrienoic acid (DHET) metabolites [44].

**Figure 1:**
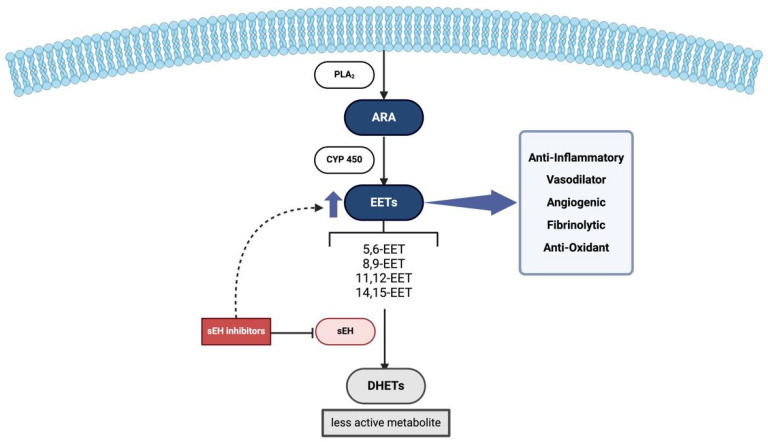
Physiological Role of EETs and sEH. Firstly, ARA is cleaved from phospholipid membranes by PLA2 within the intracellular compartment. Then EETs are produced from ARA via cytochrome P450 enzymes, exerting their anti-inflammatory and vasodilatory effects. sEH converts EETs into less active DHETs, reducing these protective effects. Inhibition of sEH will lead to increased EET levels, enhancing their beneficial roles. Abbreviations: PLA2: Phospholipase A2, ARA: Arachidonic acid, EETs: Epoxyeicosatrienoic acids, sEH: Soluble epoxide hydrolase, DHETs: Dihydroxyeicosatrienoic acids.

EETs and other EpFAs have many biological activities, including regulation of blood pressure, vasodilation, anti-inflammatory, anti-apoptotic, and antioxidant effects, as well as roles in angiogenesis, fibrinolysis, and pain perception. Among these, their anti-inflammatory properties are considered particularly significant [47]. EpFAs can reduce inflammation by blocking the nuclear translocation of nuclear factor kappa-light-chain-enhancer of activated B cells (NF-κB), thereby downregulating several proinflammatory enzymes and blocking the subsequent transcription of inflammatory cytokines [48]. Inhibiting NF-κB–mediated transcription of tumor necrosis factor alpha (TNFα) limits the recruitment of pro-inflammatory cells and the expression of vascular cell adhesion molecule-1 (VCAM-1), thus suppressing inflammation and regulating monocyte chemotaxis [49]. However, the anti-inflammatory potential of EETs is limited because sEH quickly inactivates them. EET levels can increase and begin to exhibit anti-inflammatory properties if sEH is inhibited. This mechanism is summarized in Figure 1.

In the central nervous system (CNS), EETs and other EpFAs play an important role in several pathologies through their ability to regulate cerebral blood flow, angiogenesis, and inflammation by influencing signal transduction. Since sEH is highly expressed in different regions of the brain, research on neurological conditions such as epilepsy, ischemic stroke, Alzheimer’s disease, Parkinson’s disease, and traumatic brain injury suggests that sEH inhibition can increase the levels of EETs and other EpFAs that have protective effects. EETs can prevent cell death and shrink the infarct in ischemic stroke patients. The anti-inflammatory effects of EETs can also be utilized for the prevention and treatment of seizures. Furthermore, EETs may improve mitochondrial function and reduce oxidative stress-induced neuroinflammation, and it has been demonstrated that after traumatic brain injury, genetic deletion of sEH reduces neuronal mortality, apoptosis, brain edema, and blood–brain barrier (BBB) permeability. Therefore, sEH inhibition is an important therapeutic target in the treatment of CNS diseases [25].

In particular, 14,15-EET causes blood vessel relaxation by modulating the cyclic adenosine monophosphate (cAMP)-dependent activation of protein kinase (PKA) and influencing the ADP-ribosylation of the G protein alpha subunit (Gαs) signaling pathway. This, in turn, promotes K^+^ efflux and membrane hyperpolarization via activation of large-conductance voltage- and Ca^2+^-activated K^+^ channels (BKCa) in vascular smooth muscle cells. On the other hand, sEH-mediated hydrolysis of EETs into DHETs has a negligible impact on vasorelaxation. Thus, suppressing sEH to stabilize EET levels is a potential treatment approach for hypertension management [12].

**Figure 2:**
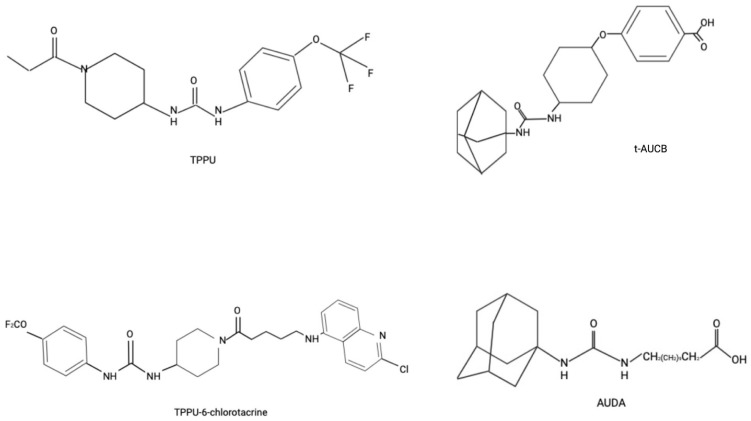
Chemical Structure of Some sEH Inhibitors.

Given their promising role in cytoprotective and anti-inflammatory properties, the development of sEH inhibitors has been actively pursued. Starting with urea-based compounds such as 12-(1-adamantylcarbamoylamino)dodecanoic acid (AUDA), 4-[4-(1-adamantylcarbamoylamino)cyclohexyl]oxybenzoic acid (t-AUCB), 1-(1-propanoylpiperidin-4-yl)-3-[4-(trifluoromethoxy)phenyl]urea (TPPU) [12], along with the newly developed and tested (1R,3S)-N-[[4-cyano-2-(trifluoromethyl)phenyl]methyl]-3-[[4-methyl-6-(methylamino)-1,3,5-triazin-2-yl]amino]cyclohexane-1-carboxamide (GSK2256294) [50,51,52]. Furthermore, multitarget inhibitors have been developed to simultaneously target various components of the arachidonic acid pathway, including cyclooxygenase-2 (COX-2) and 5-lipoxygenase (5-LOX), as well as key regulators of inflammation such as fatty acid amide hydrolase (FAAH), peroxisome proliferator-activated receptor gamma (PPARγ), farnesoid X receptor, and Raf-1 proto-oncogene serine/threonine kinase (c-RAF) [12]. Chemical structures of some of the unique sEH inhibitors are shown in Figure 2.

These advancements have translated into therapeutic prospects in the management of a variety of conditions, including insulin resistance [53], obesity [54], Kawasaki disease [55], neuropathic pain [56], subarachnoid hemorrhage (SAH) [52], intracerebral hemorrhage [57], as well as spinal cord injury [58] among others.

## 4. Alzheimer’s Disease Pathogenesis and Relation to Epoxide Hydrolase

Alzheimer’s disease is a neurodegenerative condition characterized by memory loss, cognitive decline, and brain atrophy. Aβ plaques and neurofibrillary tangles (NFTs) accumulation are the hallmarks of Alzheimer’s disease. However, the pathogenesis of Alzheimer’s disease is extremely complex and involves multiple pathways that serve as potential treatment targets [59].

Alzheimer’s disease is linked to several genetic mutations, including amyloid precursor protein (APP) and presenilin genes (PSEN1 and PSEN2) mutations that have been found to be the most common, leading to early-onset Alzheimer’s disease. Alterations in the apolipoprotein E (APOE) gene are associated with sporadic Alzheimer’s disease, where they cause changes in Aβ aggregation and clearance, leading to a higher risk of deposition [59,60]. Additionally, protein tyrosine kinase 2 beta (PTK2B), clusterin, and cholinergic receptor nicotinic alpha 2 (CHRNA2) genes are considered genetic risk factors for late-onset Alzheimer’s disease [61].

**Figure 3:**
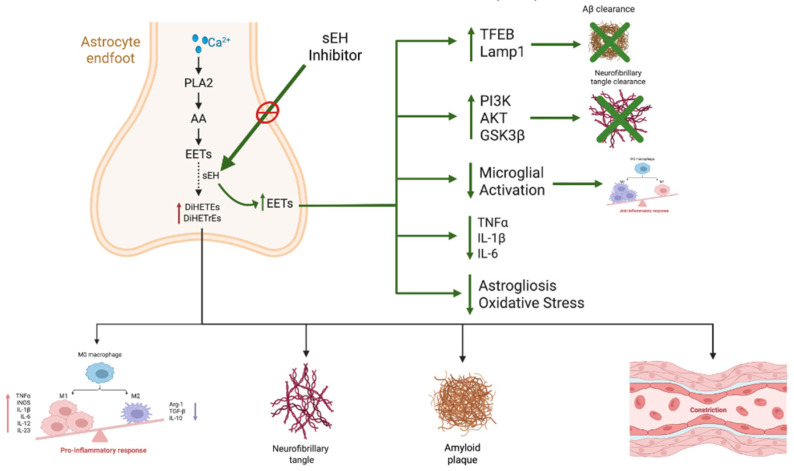
The role of epoxide hydrolase (sEH) inhibitors in the pathogenesis of Alzheimer’s disease pathology. AA, released via PLA2 from astrocytes, is metabolized into EETs, which exert anti-inflammatory and vasoprotective effects. sEH hydrolyzes EETs into diols (DiHETrEs and DiHETEs), which are pro-inflammatory. sEH inhibitors stabilize EETs, leading to: (1) activation of TFEB and LAMP1, thereby enhancing Aβ clearance; (2) modulation of the PI3K/AKT/GSK3β pathway, limiting tau hyperphosphorylation; (3) suppression of microglial activation and pro-inflammatory cytokines (TNF-α, IL-1β, IL-6), thus reducing neuroinflammation; and (4) attenuation of astrogliosis and oxidative stress, thereby promoting neuroprotection. Collectively, these actions help mitigate neurofibrillary tangle formation, amyloid plaque accumulation, vascular dysfunction, and cognitive decline. Abbreviations: PLA2—phospholipase A2; AA—arachidonic acid; EETs—epoxyeicosatrienoic acids; DiHETrEs—dihydroxyeicosatrienoic acids; TFEB—transcription factor EB; LAMP1—lysosomal-associated membrane protein 1.

Normally, amyloid precursor protein, a transmembrane protein, plays a crucial role in neuronal growth and repair and is cleaved by alpha-secretase within the Aβ region, producing soluble amyloid precursor protein (sAPPα), a nontoxic molecule. However, in Alzheimer’s disease, amyloid precursor protein is cleaved by beta-secretase, producing toxic sAPPβ, which aggregates and forms Aβ plaques [62,63]. The accumulation of Aβ plaques leads to synaptic loss, neuronal loss, and tau hyperphosphorylation [64]. Upon hyperphosphorylation, tau protein, a microtubule-associated protein, leads to the formation of neurofibrillary tangles, resulting in microtubule instability with impaired neuronal transport and function [59,60]. In addition, the accumulation of Aβ plaques and neurofibrillary tangles activates microglia and astrocytes, inducing chronic neuroinflammation. Driven by the release of cytokines such as interleukin-1β (IL-1β), interleukin-6 (IL-6), and tumor necrosis factor-α (TNF-α), neuroinflammation exacerbates neuronal damage and promotes the accumulation of amyloid-β (Aβ) plaques and neurofibrillary tangles, ultimately contributing to neuronal loss [60]. Several signaling pathways, including nuclear factor-κB (NF-κB), p38 mitogen-activated protein kinase (MAPK), and the protein kinase B (AKT)/mechanistic target of rapamycin (mTOR) axis, have been implicated in microglial activation [65]. Inflammatory cytokines such as IL-1β and TNF-α further contribute to neuronal injury by promoting tau hyperphosphorylation and Aβ aggregation [65]. Given these effects, neurodegeneration and synaptic and neurotransmitter dysfunction, particularly involving acetylcholine and glutamate, occur as key factors in the progression of Alzheimer’s disease. Impaired autophagy, along with increased oxidative stress and mitochondrial dysfunction, leads to the destruction of lipids, proteins, and DNA, disrupting cellular function and integrity [10,59]. Alzheimer’s disease is also associated with reduced cerebral blood flow and blood–brain barrier dysfunction, which exacerbates neuroinflammation and amyloid accumulation [60]. Additionally, Abnormal metabolic pathways related to lipids, insulin, and glucose further contribute to disease progression. Finally, imbalances in gut microbiota have also been linked to Alzheimer’s disease [10]. A summary of the pathogenesis and the role of sEH inhibitors in Alzheimer’s disease is shown in Figure 3.

sEH is an essential enzyme that is involved in the pathogenesis of Alzheimer’s disease. Genetic and human brain proteomic data have linked EPHX2, the gene encoding sEH, to Alzheimer’s disease [66], especially the rs7341557 variant, which is now considered an Alzheimer’s disease genetic risk factor [67]. In addition, high EPHX2 levels in the blood or brain are linked to hippocampal volume and Alzheimer’s disease progression in humans [68]. Similarly, sEH enzyme levels were observed to be elevated in murine models of Alzheimer’s disease [69,70]. The expression of EPHX2 was also found to be modified by other Alzheimer’s disease-related factors, such as the activity of the rs2279590 variant of the clusterin (CLU) gene [71], as well as obesity [72], a recognized risk factor for dementia [73].

These observations may be explained by several underlying mechanisms. Notably, elevated sEH expression has been linked to increased production of pro-inflammatory cytokines, including interleukin-1β (IL-1β) and tumor necrosis factor-α (TNF-α), as well as enhanced amyloid plaque formation and tau protein hyperphosphorylation [74,75,76].

One key mechanism involves activation of the NF-κB pathway and the NOD-like receptor family pyrin domain-containing 3 (NLRP3) inflammasome, both of which are strongly implicated in Alzheimer’s disease (AD) and related dementias [77,78]. Hydrolysis of protective epoxy fatty acids (EpFAs) by sEH removes their inhibitory effect on NF-κB signaling, thereby promoting transcription of pro-inflammatory mediators such as TNF-α, IL-1β, and IL-6. This pro-inflammatory milieu facilitates NLRP3 inflammasome activation in microglia, promotes IL-1β and IL-18 production in these cells, further amplifying neuroinflammation and accelerating neuronal injury [77]. Pharmacological inhibition of sEH has been shown to suppress NF-κB nuclear translocation and NLRP3 activation, reducing cytokine release and attenuating glial reactivity [77,78].

Additionally, the deposition of monomeric C-reactive protein (mCRP) in the brain has been directly linked to amyloid and tau pathology, and consequently, cognitive dysfunction and Alzheimer’s disease development [77]. Another study demonstrated that exposure of microglial cells to monomeric C-reactive protein (mCRP) induces morphological alterations and promotes a pro-inflammatory phenotype. This response is characterized by increased inducible nitric oxide synthase (iNOS) production and the activation of pathways such as the NLRP3 inflammasome and cyclooxygenase-2 (COX-2) [77].

Beyond inflammation, sEH contributes to mitochondrial dysfunction and impaired ROS regulation. Loss of EpFA signaling due to sEH activity leads to mitochondrial depolarization, decreased ATP production, and excessive ROS accumulation [79,80]. These processes compromise neuronal survival and synaptic integrity. Conversely, sEH inhibition preserves mitochondrial function, restores membrane potential, reduces lipid peroxidation, and enhances antioxidant defenses such as nuclear factor erythroid 2-related factor 2 (Nrf2) and superoxide dismutase (SOD1) [78,81].

sEH also intersects with metabolic regulators critical for brain health, including AMP-activated protein kinase (AMPK). AMPK, a master regulator of cellular energy balance, is suppressed under chronic inflammation and oxidative stress, impairing autophagy and neuronal resilience [82]. By stabilizing EpFAs, sEH inhibitors indirectly enhance AMPK activity, promoting mitochondrial biogenesis, autophagic clearance of amyloid-β, and synaptic protection [82].

These findings can be attributed to the role of sEH, which metabolizes anti-inflammatory and vasodilatory epoxides such as epoxyeicosatrienoic acids (EETs) and epoxydocosapentaenoic acids (EDPs). Both EETs and EDPs are derived from arachidonic acid (ARA) and are synthesized by epoxygenases. In the murine brain, 14,15-EET is the predominant regioisomer in the cortex, whereas 11,12-EET is more abundant in the cerebral, cerebellar, and hippocampal regions [83]. These molecules have been implicated in many Alzheimer’s disease-related findings. 14,15-EET exerts a protective function against Alzheimer’s disease pathology [84], since lower concentrations were observed in the hippocampus and cortex of Alzheimer’s disease mouse models compared to wild-type controls. Furthermore, treatment of Alzheimer’s disease mouse models with 14,15-EET has demonstrated prevention of disease progression by promoting the clearance of Aβ plaques in astrocytes. This effect is mediated by enhanced astrocytic lysosomal function through activation of lysosomal-associated membrane protein 1 (LAMP1), regulated by the transcription factor EB (TFEB). A study showed that the application of 14,15-EET to mouse brains led to nuclear translocation of TFEB from the cytoplasm and stimulation of the lysosomal calcium channel transient receptor potential mucolipin 1 (TRPML1). This, in turn, promoted lysosomal activity and Aβ clearance. Importantly, blocking TRPML1 reduced astrocytic Aβ clearance, confirming the pathway’s involvement. This effect was also replicated by deletion of the EPHX2 gene, which increases endogenous EET levels [61]. Additionally, in vitro incubation of mouse brain tissue with Aβ resulted in lower cerebral levels of 14,15-EET & dihydroxyeicosatetraenoic acid (DiHETE) and astrocytic and neuronal 11,12-EET & DiHETE, specifically in the hippocampus [83]. Substrates of soluble epoxide hydrolase (sEH), including epoxyeicosatetraenoic acids (EEQs) and epoxydocosapentaenoic acids (EDPs), have been reported to enhance amyloid-beta (Aβ) clearance by macrophages. In addition, these lipid mediators influence inflammatory cytokine levels and help regulate endoplasmic reticulum (ER) stress by activating signaling pathways involving X-box binding protein 1 (XBP1), activating transcription factor 4 (ATF4), and activating transcription factor 6 (ATF6) [85]. Treatment with 11,12-EET was also found to enhance microglial uptake and clearance of tau protein in mice [70].

Moreover, a study conducted on a sample of Alzheimer’s disease patients demonstrated connections between sEH-related metabolites found in plasma and cerebrospinal fluid (CSF), and the development of the disease. The study revealed that 17,18-DiHETE had plasma levels that were 3 times greater in Alzheimer’s disease patients in comparison to controls [86]. Also, an association between 11,12 dihydroxyicosatrienoic acids and 14,15 dihydroxyicosatrienoic acids (DiHETrE), as well as docosahexaenoic acid derivative 19,20-DiHDPA, was found with specific cerebrospinal fluid (CSF) proteins. Higher levels of these metabolites were correlated with higher levels of proteins responsible for the regulation of vascular function. However, they were negatively associated with proteins related to cell proliferation, energy, and glucose metabolism, which are key pathways implicated in Alzheimer’s disease [87]. Another study revealed that higher levels of other sEH metabolites, such as 14,15 DiHETE and 19,20 DiHDPA, were detected in the plasma of human females with Alzheimer’s disease and males with either Alzheimer’s disease or mild cognitive impairment, compared to cognitively normal individuals. This relationship was even more pronounced in individuals carrying the ApoE4 variant, especially those with existing amyloid and tau pathology. These findings highlight a potential interaction between sex, genetics, and the sEH enzyme [88].

In addition, hepatic sEH metabolizes anti-inflammatory epoxy fatty acids into pro-inflammatory diols such as DiHETrEs and DiHDPA, which have been found to be elevated in the plasma and CSF of AD patients. These diols circulate systemically and influence vascular tone, systemic inflammation, immunity, and BBB permeability, thereby creating a proinflammatory environment that contributes to neurodegeneration [76,88].

Interestingly, an elevation of mEH expression in the brain specimens from Alzheimer’s disease patients was observed, predominantly in the hippocampal and cortical astrocytes and neurons surrounding Aβ plaques [32]. Finally, higher levels of expression of the EPHX4 gene were linked to improved cognitive resilience in older adults and reduced Alzheimer’s disease pathology [89].

### 4.1. Use of Soluble Epoxide Hydrolase (sEH) Inhibitors in Alzheimer’s Disease

The role of sEH inhibitors has gained attention as they target several pathogenic pathways in Alzheimer’s disease. This growing focus has translated into multiple studies investigating the impact of sEH gene deletion on the development and progression of the condition. sEH gene deletion was associated with an improvement in Alzheimer’s disease-related behaviors in mice, such as nest building and spatial memory [90]. In addition, these mice exhibited reduced concentrations of cortical and hippocampal Aβ monomers and oligomers, as well as ApoE protein. Furthermore, sEH deletion was associated with higher levels of IL-4 and IL-10, which are anti-inflammatory cytokines. This was linked to exacerbated astrogliosis, pro-inflammatory cytokines, NF-κB function, and nuclear factor of activated T cells (NFAT) activation. Finally, experiments revealed the regulation of several Alzheimer’s disease-associated biological pathways in mice with the deleted gene, including astrocytic activation and pro-inflammatory cytokine production, which led to the activation of the STAT3 pathway around Aβ plaques [91].

Another study [61] exploring the impact of EPHX2 deletion in a murine model of Alzheimer’s disease revealed lower levels of Aβ deposition in the hippocampus and motor cortex of these mice. Interestingly, this change was not associated with amyloid precursor protein (APP) production; rather, it was likely due to the prevention of insoluble Aβ plaque formation. Behavioral and cognitive improvement, microglial phagocytosis, and reduced astrocytic activation mediated via 14,15-EET and lysosomal biogenesis pathways were observed with increased clearance of Aβ plaques. Similarly, EPHX2 gene deletion in Alzheimer’s disease mouse models resulted in reduced tau pathology [70], further supporting the potential role of sEH in Alzheimer’s disease pathology.

These findings, coupled with the previously established anti-inflammatory, antioxidant [18], and neuroprotective role of EETs [18,74], which can be preserved through sEH inhibitors, have highlighted the need to further investigate the potential of such inhibitors. In concurrence with this, studies have shown EPHX2 to be a targetable gene for Alzheimer’s disease management, without potential side effects [68]. A summary of the pathogenesis and the role of sEH inhibitors in Alzheimer’s disease is shown in Figure 3.

Several compounds have been formulated and tested for this purpose. The following are some key drugs and their respective functions in Alzheimer’s disease:

#### 4.1.1. TPPU

1-(1-propanoylpiperidin-4-yl)-3-[4-(trifluoromethoxy)phenyl]urea is the most extensively studied compound that is a potent and selective inhibitor of sEH in both primates and rodents. It has high target occupancy with slow and tight-binding inhibition [92] and good penetration of the blood–brain barrier. TPPU has a half-maximal inhibitory concentration (IC50) of human sEH of <50 nM (3.7 nM) [13]. In addition, it is a selective inhibitor of the p38β and p38γ isoforms of p38 mitogen-activated protein kinase (p38 MAPK), which have been linked to Alzheimer’s disease. Targeting both sEH and p38β simultaneously may elicit synergistic neuroprotective effects. This dual approach also contributes to the suppression of NF-κB activation, a key mediator of neuroinflammation [92].

The utilization of behavioral testing, such as novel object recognition (NOR), object location test (OLT), open field test (OFT), and Y-maze, has consistently demonstrated improvements in memory, cognition, and spatial learning in various TPPU-treated mouse models [61,69,70,78,79,81,92,93,94,95,96,97]. Behavioral improvements have also been observed in transgenic Drosophila, which had enhanced crawling ability and olfactory memory following treatment with TPPU [81].

TPPU’s ability to enhance memory preservation possibly comes from its ability to reduce amyloid precursor protein (APP) expression and Aβ plaque formation, particularly in the medial cortex and hippocampus of 5×FAD mice [61,78,92,93,94,95,97]. One of the mechanisms achieving this is through decreased expression of the receptor for advanced glycation end-products (RAGE), which facilitates the influx of Aβ into the brain, and by increasing the levels of ATP-binding cassette subfamily B member 1 (ABCB1/P-glycoprotein), which functions as an efflux pump clearing Aβ from the brain [69]. TPPU administration was also found to enhance Aβ clearance by upregulating the expression of angiotensin-converting enzyme (ACE) and insulin-degrading enzyme (IDE), all of which are involved in the degradation of Aβ plaques [85]. Additionally, it was found to also reduce *p*-tau levels and tau hyperphosphorylation. This effect is likely mediated through the reduction of Aβ plaques and subsequent inhibition of p38 MAPK activation via RAGE and nicotinic acetylcholine receptor signaling. TPPU was also found to regulate the phosphatidylinositol 3-kinase (PI3K)/AKT/Glycogen synthase kinase-3β (GSK-3β) pathway, suppress oxidative stress, and enhance microglial clearance by maintaining epoxyeicosatrienoic acid (EET) levels, all of which contribute to the reduction of tau pathology [70,78,92,93,94,96,98].

TPPU has demonstrated its ability to reduce neuroinflammation by stabilizing EETs, thereby enhancing and prolonging their anti-inflammatory effects [61,78,81,92,93,94,95,97]. Some of the main pathways that TPPU modulates include the p38 MAPK/NF-κB, Toll-like receptor 4 (TLR4)/myeloid differentiation primary response gene 88 (MyD88)/NF-κB, and NLRP3 pathways, along with their related cytokines, including COX-2, *p*-p65/p65, IL-6, inhibitor of nuclear factor kappa B kinase subunit beta (*p*-IKKβ/IKKβ), and IL-18. It was found that TPPU could stop the Aβ plaques-induced activation of these pathways [70,78,81,92]. It also reduces the levels of many other proinflammatory cytokines, such as TNF-α, CC chemokine ligand (CCL-2), CXC chemokine ligand (Cxcl-1), IL-23A, and iNOS [77,85,95]. The anti-inflammatory action of TPPU was also noted in flies, leading to a decrease in TNF and IL-1 concentrations [81].

Furthermore, studies have demonstrated that TPPU exerts its effects by attenuating astrocyte activation, as evidenced by reduced expression of the astrocytic marker glial fibrillary acidic protein (GFAP). In addition, it suppresses microglial activation, reflected by decreased levels of microglial markers such as ionized calcium-binding adaptor molecule 1 (Iba1) and triggering receptor expressed on myeloid cells 2 (Trem2), ultimately leading to a reduction in neuroinflammation [61,69,70,78,81,93,94,95]. It also restores normal microglial morphology and COX-2 levels, supports homeostatic microglia, while reducing disease-associated microglia (DAM) and interferon-responsive microglia (IFN). This helps regulate pathways linked to cytokine production, immune responses, lipid metabolism, and lysosomal activation, as revealed by gene expression analysis [70]. TPPU also stimulates polarization towards anti-inflammatory M2 microglia, evident through CD206 and suppressor of cytokine signaling 3 (SOCS3) markers [81].

In addition, TPPU was shown to be effective in the reduction of oxidative stress and ER stress, through preserving mitochondrial function [92], glutathione levels [78], and clearing reactive oxygen species [79]. Furthermore, it restores mitochondrial membrane potential, enhances ATP production, and reduces mitochondrial ROS in 5xFAD mouse models. Those mitochondrial benefits were associated with enhanced memory and preserved synaptic integrity, emphasizing the role of sEH inhibitors in neuroprotection [80]. It also contributes to preventing lipid peroxidation [81] and displays down-regulation of glucose-regulated protein 78 (GRP78), kelch-like ECH-associated protein 1 (keap1), Aldehyde Oxidase 1 (Aox1), and MDA. Conversely, it upregulates heme oxygenase 1 (HO-1), nuclear factor erythroid 2-related factor 2 (Nrf2), and superoxide dismutase 1 (SOD1), all of which play key roles in alleviating endoplasmic reticulum (ER) stress [13,78,81].

Moreover, TPPU was found to have a role in CBF improvement, evident through enhanced neurovascular coupling, which is the mechanism by which blood flow increases in response to neuronal activity. This effect was mediated by elevated production of endothelial nitric oxide synthase (eNOS) in the hippocampi of mice. TPPU additionally led to arteriolar dilation in vitro and reduced cortical and hippocampal capillary loss, demonstrated through increased expression of desmin and α-smooth muscle actin (α-SMA), which are capillary pericyte protein markers. It also reduced capillary leakage, thereby decreasing the risk of cerebral inflammation [69]. TPPU was additionally shown to enhance the myogenic tone and contractile function of cerebral vascular smooth muscle cells (VSMCs), thereby improving the autoregulation of cerebral blood flow (CBF) [79].

Furthermore, TPPU demonstrates a neuroprotective function by preserving neuronal activity and structure, enhancing synaptic plasticity and glutamatergic function evident through an increased number of pre- and postsynaptic markers, and limiting hippocampal neuron loss in Alzheimer’s disease models [61,69,70,78,81,94,95]. This was demonstrated through anti-apoptotic activity through upregulation of BCL2 apoptosis regulator, and downregulation of bcl-2-like protein-4, p53, and caspases [78]. TPPU has shown protective effects on Human microglial clone 3 (HMC3) cells as well [81]. It also induced epigenetic modifications characterized by increased levels of DNA methylation and hydroxymethylation, along with a reduction in the expression of histone deacetylases Hdac1 and Hdac2 in the brain. These changes were associated with improved cognitive function [93].

Finally, transcriptome analyses have been performed to determine the effects of TPPU administration on differential gene expression. The analyses revealed changes in genes involved in a variety of functions, including glutamate transport and excitation, Aβ clearance, oxidative stress, inflammation, angiogenesis, synaptic function, cytoskeletal and axonal integrity, and cellular contractility [69,70,79]. These findings support the mechanisms mentioned earlier in this review and suggest that the effects of TPPU against Alzheimer’s disease pathology are mediated through multiple pathways.

#### 4.1.2. UB-SCG-51

4-[4-[(12-chloro-10-tetracyclo[8.3.1.18,12.02,7]pentadeca-2,4,6-trienyl)carbamoylamino]cyclohexyl]oxybenzoic acid (UB-SCG-51) is a novel sEH inhibitor derived from Benzohomoadamantane [99]. It is a selective and potent inhibitor that has demonstrated anti-inflammatory effects with no toxicity in human neuroblastoma and microglial cell lines, while only minimal astrocyte toxicity was observed at high concentrations. This compound was evaluated in murine models of Alzheimer’s disease following oral administration. It showed effective blood–brain barrier penetration and achieved therapeutic concentrations within the brain. Notably, treatment resulted in improved memory and cognitive performance measured by OFT, OLT, and NORT tests, which may be attributed to a marked reduction in Aβ plaque deposition. This effect was mediated by the upregulation of Aβ-degrading enzymes, including insulin-degrading enzyme (IDE) and neprilysin. Additionally, this compound reduced phosphorylated tau aggregates, which was associated with decreased cyclin-dependent kinase 5 (CDK5) expression. Additionally, hippocampal Iba-1 levels and GFAP activity were reduced, which are correlated with less microglial reactivity and astrogliosis, respectively [100].

UB-SCG-51 administration has been shown to lower the expression of pro-inflammatory cytokines, namely Il-1β, CCL3, CCL12, and Trem2, which are mediators of glial activation and contribute to Alzheimer’s disease progression. In a murine model of Alzheimer’s disease, this molecule has also shown neuroprotection through enhancing the expression of genes correlated with neuroplasticity and reducing levels of NF-κB. Furthermore, it is associated with decreased levels of Calpain-1, Caspase-3, and higher levels of brain-derived neurotrophic factor (BDNF), which are associated with reduced neuronal death [100].

#### 4.1.3. UB-SCG-74

UB-SCG-74, an arginine salt of UB-SCG-51, is a selective, potent, and safe sEH inhibitor with good oral absorption and BBB permeability. In mice, improved working and spatial memory, as well as enhanced synaptic plasticity and dendritic density, were found to be superior to those achieved with donepezil. Additionally, these effects were observed to last for 4 weeks, making this compound a promising candidate for human studies [101].

#### 4.1.4. UB-SCG-55

UB-SCG-55 is another safe, non-cytotoxic, and potent benzohomoadamantane sEH inhibitor that was found to decrease the expression of iNOS as well as nitric oxide (NO) release in mCRP-treated mice microglial cells [77]. It reduces the activation of NLRP3 and COX-2 inflammatory pathways, with a significant decrease in proinflammatory cytokines, TNF-α, IL-6, and C-C motif chemokine ligand (CCL). In primary human microglia, it reverts the M1 proinflammatory state to normal morphology [77].

#### 4.1.5. UB-BJ02

UB-BJ02 is a novel sEHi that has significantly reduced amyloid beta (Aβ) plaque burden and suppressed activation of hippocampal astrocytes and microglia, leading to decreased expression of pro-inflammatory cytokines such as IL-6, TNF-α, IL-18, and IL-1β in mice. These changes translated into improved performance in NORT and OLT tests [102]. UB-BJ02 also modulated Alzheimer’s disease risk molecules by decreasing the expression of triggering receptor expressed on myeloid cells 2 (TREM2) and increasing cluster of differentiation 33 (CD33). Additionally, it influenced mitochondrial function by reducing dynamin-related protein 1 (DRP1) and phosphatase and tensin homolog (PTEN), which are markers of mitochondrial fission and apoptosis, while enhancing mitochondrial fusion protein optic atrophy 1 (OPA1) and biogenesis mediator peroxisome proliferator-activated receptor gamma coactivator 1-alpha (PGC-1α) [102]. Importantly, UB-BJ02 improved gut microbiota diversity in 5xFAD mice and exerted anti-inflammatory effects in both the gut and spleen. It increased the abundance of beneficial microbial species like Lactobacillus intestinalis, Staphylococcus nepalensis, and Prevotella sp004792655, which are negatively associated with amyloid plaque accumulation and positively correlated with cognitive function [102].

#### 4.1.6. TPPU-6-Chlorotacrine/TPPU-Huprine Y

Acetylcholinesterase inhibitors (AChEIs) are a mainstay in Alzheimer’s disease treatment, as they enhance cholinergic transmission and cognitive function [103]. Recently, dual sEH/AchE inhibitors have been synthesized for targeting Alzheimer’s disease by linking 6-chlorotacrine or huperine Y, a chiral derivative of tacrine, to the propanoyl group of TPPU [104]. Multiple hybrids have been synthesized and tested, demonstrating potent and stable in vitro inhibition of human sEH and AChE enzymes, with adequate CNS penetration and blood–brain barrier permeability, all while maintaining low toxicity. Furthermore, in vivo administration to Alzheimer’s disease mouse models resulted in better memory, reduced expression of inflammatory compounds (NF-κB, IL-6, IL-1β, and GFAP), and improved synaptic plasticity, mediated by synaptophysin. Greater levels of sAPPα and reduction of sAPPβ levels and tau phosphorylation at the Ser396 site have also been reported [104].

It is worth noting that coadministration of TPPU with acetylcholinesterase (AChE) inhibitors, namely 6-chlorotacrine and rivastigmine, enhances the efficacy of TPPU by improving working memory and cognitive function in mice, lowering IL-6, Trem2, and GFAP expression while elevating the levels of synaptophysin (SYN), cyclic AMP-responsive-element-binding protein (*p*-CREB/CREB) ratio, and brain-derived neurotrophic factor (BDNF). This signifies their role in improving plasticity and synaptic function [94].

#### 4.1.7. AS-2586114 and UB-EV-52

AS-2586114 [3-cyclopropyl-4-[4-(2-[(1S,2R,5S)-6,6-dimethylbicyclo(3.1.1) heptan-2-yl] methylamino)-2-oxoethyl]piperidin-1-yl]benzoic acid monohydrochloride] is a recently explored sEH inhibitor with an IC50 for human sEH of 0.4 nM. In vivo, it has prolonged activity and BBB crossing ability [13]. UB-EV-52 [4-[4-(2-oxatricyclo[3.3.1.13,7]decan-1-ylcarbamoylamino)cyclohexyl]oxybenzoic acid] is a newer agent related to adamantane-derived sEH inhibitors. It has excellent solubility (>100 μM) and good microsomal stability. In addition, it can cross the BBB and is minimally inhibited by the cytochrome system [13].

In vitro human tissue studies, along with studies conducted in mice using these two compounds and TPPU, have demonstrated potent inhibition of brain sEH with no cytotoxicity. Administering these inhibitors reduced the release of hippocampal inflammatory cytokines, such as TNF-α, IL-1β, and CCL3, and decreased oxidative stress by lowering hydrogen peroxide levels. Additionally, these inhibitors reduced the expression of genes associated with oxidative stress, such as HO-1, aldehyde oxygenase 1 (AOX1), and superoxide dismutase 1 (SOD1). They also lowered serine/threonine-protein kinase/endoribonuclease inositol-requiring enzyme 1 α (IRE1α), ATF-6, and XBP1, indicating reduced endoplasmic reticulum stress. Reduced Aβ accumulation and sAPPβ and C-terminal fragments (CTFs) production have also been observed. Working memory was also improved, and importantly, these sEH inhibitors showed superior memory preservation effects when compared to donepezil [13].

The benefits of sEH inhibitors are primarily attributed to their direct central effects, with agents like TPPU demonstrating blood–brain barrier (BBB) permeability and modulating neuroinflammation, synaptic loss, and amyloid deposition. However, peripheral inhibition may also influence systemic inflammation, thereby indirectly improving brain pathology [61,92].

#### 4.1.8. Other Inhibitors

A study evaluated the efficacy of 6 candidate sEH inhibitors (UB21, UB23, UB24, UB28, EPB50, and EV52) synthesized from varying structural families, with TPPU as a control [105]. Of these, EV52, UB24, and UB21 demonstrated cytotoxic effects in the human neuroblastoma cell lines, whereas UB23 and UB28 showed potential anti-inflammatory effects in BV2 microglial cell lines by decreasing nitric oxide release [105].

Furthermore, 1-O-acetyl-4R,6S-britannilactone (AB) is a natural sEHi isolated from the flowers of Inula britannica var. chinensis, known for their anti-inflammatory effects, particularly in gastrointestinal and pulmonary conditions [106]. AB has been shown to reduce microglial activation and inflammation while enhancing autophagy, mainly through the activation of the Mitogen-Activated Protein Kinase (MAPK) and AMP-Activated Protein Kinase (AMPK) pathways. Additionally, AB binds to PDZ-binding kinase (PBK), thereby inhibiting the degradation of the anti-inflammatory protein TNF-α-Induced Protein 2 (TIPE2). This is achieved by preventing its Serine 3 (Ser3) phosphorylation-mediated ubiquitination. It was found that this mechanism leads to anti-inflammatory effects similar to those observed with PBK knockout in mice models [107].

Finally, the phosphatase activity of soluble epoxide hydrolase (sEH) has emerged as a potential therapeutic target for Alzheimer’s disease. Ebselen, a compound with established antioxidant and anti-inflammatory properties, has been shown to inhibit the phosphatase function of sEH. Notably, treatment with ebselen led to a reduction in Aβ plaque formation and tau phosphorylation levels in experimental models [108]. These effects may, at least in part, be mediated through the inhibition of sEH [109]. The role of sEH inhibitors and their use in Alzheimer’s disease is summarized in Table 1.

## 5. The Role of Epoxide Hydrolase Inhibitors in Cognitive Impairment/Decline in Other CNS Disorders

### 5.1. Parkinson’s Disease

Parkinson’s disease (PD) is one of the most common neurodegenerative diseases affecting more than 5 million people worldwide [163]. It is characterized by aggregations of α-synuclein protein and loss of dopaminergic neurons [164]. To date, all available medications only offer symptomatic treatment, and there are no therapies to cure or stop the progression of the disease. Multiple studies have shown that oxidative stress linked to neuroinflammation plays a role in the pathophysiology of Parkinson’s disease [165,166]. Postmortem brain samples have exhibited oxidative stress, inflammation, and mitochondrial dysfunction in the affected brain regions of Parkinson’s disease patients [167].

Investigations into the role of soluble epoxide hydrolase (sEH) in Parkinson’s disease (PD) have provided compelling evidence for its involvement in the pathogenesis of the disease. The use of the potent sEH inhibitor TPPU and genetic deletion of sEH in mice demonstrated a protective effect against 1-methyl-4-phenyl-1,2,3,6-tetrahydropyridine (MPTP)-induced neurotoxicity in dopaminergic neurons and revealed a reduction in oxidative stress in the striatum. On the other hand, overexpression of sEH has been linked to increased neurotoxicity [117] and levels of phosphorylated α-synuclein, a marker of PD pathology [117]. Postmortem analysis of brain samples from patients with dementia with Lewy bodies has shown elevated sEH levels, which further supports this notion [117]. Interestingly, the study also investigated induced pluripotent stem cells (iPSCs) derived from familial Parkinson’s disease patients carrying PARK2 mutations, where TPPU-mediated inhibition of sEH effectively prevented apoptosis in dopaminergic neurons [117]. A recent study showed that kurarinone, a natural non-competitive inhibitor of soluble epoxide hydrolase (sEH), alleviates neurodegeneration in an MPTP-induced murine Parkinson’s model by increasing levels of EETs [137]. These findings emphasize the potential therapeutic significance of targeting sEH in Parkinson’s disease.

### 5.2. Vascular Dementia

Vascular cognitive impairment and dementia (VCID) is one of the leading causes of dementia [168]. One study has identified elevated levels of 14,15-DHET, an inactive metabolite of 14,15-EET, indicating altered lipid metabolism in individuals with vascular cognitive impairment (VCI) compared to age-matched controls. Immunohistochemical analysis revealed prominent sEH expression in cerebral microvascular endothelium, with elevated levels observed in areas adjacent to cerebrovascular lesions in VCI [169]. Moreover, alterations in serum oxylipin profiles, indicative of enhanced soluble epoxide hydrolase (sEH) activity, have been identified as a critical contributor to the pathophysiology of vascular cognitive impairment [170]. Therefore, sEH inhibition could be a potential intervention in vascular cognitive impairment.

The neuroprotective effects of sEH inhibition, using the specific inhibitor TPPU, were investigated in a rat model of vascular cognitive impairment induced by chronic cerebral hypoperfusion (CCH) through bilateral carotid artery stenosis (BCAS) [114]. The results indicate that chronic administration of TPPU prevents memory deficits in rats subjected to BCAS, as evidenced by improved cognitive function in a novel object recognition test. Furthermore, TPPU treatment enhances dilation in cerebral parenchymal arterioles, suggesting improved cerebral blood flow. The study also explores changes in mRNA expression related to neuroplasticity and inflammation in the brains of bilateral carotid artery stenosis (BCAS) rats treated with TPPU. It showed that treatment increases the expression of doublecortin, a marker of young neurons and neuronal precursors, as well as superoxide dismutase 3 (SOD3), an antioxidant enzyme. Finally, an increase in sEH mRNA expression was noted, indicating a potential feedback mechanism [114]. Overall, these findings suggest that sEH inhibition exerts neuroprotective effects in the context of VCID induced by chronic cerebral hypoperfusion.

### 5.3. Stroke

Stroke is the second most prevalent cause of death worldwide and leaves up to 50% of survivors with enduring disabilities [171]. Stroke causes neuroinflammation, leading to neuronal loss and activation of residual immune cells. This results in promoting the production of proinflammatory cytokines and disruption of the BBB, causing further damage [172]. Neuroprotective agents developed for cerebral ischemia have demonstrated effectiveness in animal models, but not in clinical trials [173]. Soluble epoxide hydrolase contributes to stroke pathology by metabolizing EETs, which possess anti-inflammatory effects and neuroprotective properties against cerebral ischemia [174]. Moreover, knockout of the sEH gene has demonstrated a protective effect in models of experimental cerebral ischemia, further supporting the role of sEH in the pathogenesis of stroke [175].

The sEH inhibitor AUDA possesses a variety of properties that make it a promising candidate for stroke treatment. It acts by elevating the concentration of EETs, which serve as neuroprotective and anti-inflammatory mediators. This supports the preservation of cognitive function by attenuating neuroinflammatory responses and reducing oxidative stress within the brain [176].

In a mouse model of middle cerebral artery occlusion (MCAO), AUDA-butyl ester (AUDA-BE) significantly reduced infarct size by 40–50%, with the neuroprotective effect shown to be specific to sEH inhibition. Notably, coadministration with N-methylsulfonyl-6-(2-prop-2-ynoxyphenyl) hexanamide (MS-PPOH), an inhibitor of EET biosynthesis, attenuated the protective effects of AUDA-BE [132]. In a rat model of middle cerebral artery occlusion (MCAO), administration of the sEH inhibitor AUDA induced a shift in microglial polarization toward the anti-inflammatory M2 phenotype, as evidenced by decreased CD86 (M1 marker) and increased CD206 (M2 marker) expression within the ischemic brain region. Furthermore, AUDA treatment suppressed pro-inflammatory cytokine production in the ischemic cortex while enhancing the expression of antioxidant enzymes, including catalase, heme oxygenase-1 (HO-1), and superoxide dismutase 1 (SOD1), alongside activation of the transcription factor nuclear factor erythroid 2-related factor 2 (Nrf2). This coordinated antioxidant response correlated with increased neuronal survival in the affected cortical area [133]. The n-butyl ester prodrug form of AUDA (AUDA-nBE) has a favorable pharmacokinetic profile because of its increased solubility, which enhances the drug’s bioavailability and distribution. Additionally, the active drug is released in a sustained manner and has a longer half-life in this prodrug form, thus making it a promising treatment option [177].

Administration of TPPU was shown to have anti-apoptotic, antioxidant, anti-inflammatory, and mitochondrial protective effects through the inhibition of NF-κB or p38 MAPK signaling pathways. TPPU also decreases brain edema, suggesting improved blood–brain barrier function [116]. Collectively, soluble epoxide hydrolase inhibition may ameliorate post-ischemic neurocognitive deficits by preserving neuronal function and reducing neuroinflammation.

### 5.4. Diabetes-Related Cognitive Impairment

There is growing recognition that cognitive dysfunction is a significant comorbidity of diabetes mellitus. Among these affected functions, memory and learning are primarily regulated by the hippocampus. Diabetes-related cognitive deficits are directly linked to impairments in hippocampal neurogenesis and synaptic plasticity [178]. The generation of advanced glycation end products (AGEs), a key mediator in the pathophysiology of diabetes complications, is enhanced by uncontrolled hyperglycemia. Advanced glycation end products (AGEs) cause pro-inflammatory and pro-oxidant conditions, which lead to neuronal degeneration [179]. EETs exhibit a wide range of biological activities, such as the reduction of inflammation, pathological fibrosis, apoptosis, and reactive oxygen species (ROS) in other neurological diseases [180]. Thus, sEH is a potential target in diabetes.

A study demonstrates that sEH inhibitor *t*-AUCB exhibits notable cognitive benefits in a type 2 diabetes mouse model [144]. Treatment with *t*-AUCB leads to improved learning and memory. *t*-AUCB mitigates oxidative stress in the hippocampus and reduces apoptosis by decreasing reactive oxygen species levels and altering the expression of apoptosis-related proteins. It also restores the synaptic proteins, postsynaptic density protein 95 (PSD95) and N-methyl-D-aspartate receptor subtype 2B (NR2B). Additionally, it increases the expression of proline-rich tyrosine kinase 2 (Pyk2), a nonreceptor tyrosine kinase associated with synaptic plasticity [144]. Finally, a related study showed that *t*-AUCB slows down BBB damage in diabetic mice through the activation of the AMP kinase/HO-1 pathway [82]. These findings collectively suggest that sEH inhibition with *t*-AUCB holds therapeutic promise for alleviating cognitive deficits associated with type 2 diabetes through multiple molecular mechanisms.

Another related study showed that the administration of TPPU demonstrated significant protective effects in a streptozotocin-induced diabetic rat model [181]. TPPU improved cognitive function, as shown by enhanced performance in behavioral tests assessing spatial memory and passive avoidance response. Moreover, TPPU effectively lowered fasting blood glucose levels in diabetic rats. It also ameliorated diabetes-induced alterations in neurotransmitter levels, particularly through reducing elevated gamma-aminobutyric acid (GABA) levels in the hippocampus and restoring norepinephrine levels while decreasing dopamine levels in the cerebellum. Additionally, TPPU exhibited antioxidant properties by regulating malondialdehyde and glutathione levels in the brains of diabetic rats [181]. These findings suggest that TPPU may hold potential as a therapeutic intervention against diabetes-associated cognitive impairment.

## 6. Conclusions

sEH has emerged as a critical enzyme in the pathogenesis of AD and other neurological disorders due to its role in the degradation of anti-inflammatory epoxy-fatty acids, such as EETs and EDPs. These lipid mediators have been shown to exert neuroprotective effects by reducing oxidative stress, suppressing neuroinflammation, maintaining blood–brain barrier integrity, and preserving neuronal function. Elevated sEH expression, observed in both human AD patients and murine models, has been strongly correlated with increased neuroinflammatory cytokines, Aβ plaque accumulation, and tau hyperphosphorylation, which are key pathological features of AD.

Inhibition of sEH, either pharmacologically or genetically, has demonstrated significant therapeutic potential in preclinical models. Several inhibitors, including TPPU, UB-SCG-74, and AS-2586114, have shown promising results in reducing AD-related pathology, restoring synaptic function, improving cognitive behavior, and limiting neuronal apoptosis. These effects are achieved via multiple molecular pathways, such as downregulation of NF-κB, suppression of NLRP3 inflammasome activity, modulation of the PI3K/AKT/GSK3β axis, and enhancement of lysosomal clearance mechanisms. The neurovascular benefits of sEH inhibitors, such as improved cerebral blood flow and reduced capillary leakage, further support their potential as multifaceted therapeutic agents.

Moreover, the development of dual inhibitors that combine sEH inhibition with acetylcholinesterase inhibition has shown synergistic effects, addressing both the pathological and symptomatic aspects of AD. These multitarget compounds not only modulate neuroinflammation and oxidative stress but also enhance cholinergic signaling and synaptic plasticity. Such dual-action agents could provide a more effective approach than current monotherapies, especially in the early stages of disease progression.

Importantly, sEH inhibitors extend their neuroprotective promise beyond AD. They have demonstrated beneficial effects in other central nervous system disorders, including Parkinson’s disease, vascular dementia, stroke, and diabetes-related cognitive impairment. This positions sEH as a versatile therapeutic target across a broad spectrum of neurodegenerative and neurovascular diseases.

## 7. Future Directions

Despite promising preclinical results, the clinical translation of sEH inhibitors for Alzheimer’s disease remains a major challenge. Ensuring effective blood–brain barrier (BBB) penetration, establishing long-term safety profiles, and demonstrating efficacy in human trials are critical next steps [70,101,182]. Although compounds such as TPPU and UB-SCG-74 show favorable pharmacokinetics and CNS penetration in animal models, further studies are required to confirm these findings in humans [101,182].

Designing robust clinical trials with well-defined patient populations, optimized dosing strategies, and appropriate biomarkers will be essential to assess the true therapeutic potential of these inhibitors [183]. Beyond monotherapy, investigating the potential of dual treatments involving sEH inhibitors and existing Alzheimer’s disease drugs may yield synergistic effects [94]. Additionally, recent studies have raised interest in exploring the role of hepatic sEH and the liver-brain axis in cerebral Aβ metabolism, which warrants further investigation [84].

Several sEH inhibitors, including AR9281, GSK2256294, and EC2056, have entered early-phase clinical trials in other indications, laying the groundwork for future trials in neurodegenerative conditions [50,51,52,56,184,185]. Advancing these compounds through well-structured clinical pipelines will be key to realizing their potential as disease-modifying agents in AD and related disorders.

While this review provides a focused and comprehensive overview, a few limitations should be noted. For instance, it primarily focused on sEH inhibition, without extensively addressing other potential therapeutic targets or pathways involved in Alzheimer’s disease and related neurological disorders. Additionally, the scope of this review was limited to neurological conditions and did not explore the broader systemic implications of sEH inhibition, such as its roles in cardiovascular or metabolic diseases. Finally, the literature search was conducted using a limited number of databases, which may have resulted in inadvertent exclusion of relevant studies published elsewhere.

In conclusion, epoxide hydrolase inhibitors, particularly those targeting sEH, represent a promising therapeutic approach for Alzheimer’s disease and other neurological disorders. While significant progress has been made in preclinical studies, further research is needed to translate these findings into effective treatments for patients. The multifaceted effects of sEH inhibition on neuroinflammation, oxidative stress, and protein aggregation make it an attractive target for future drug development efforts in the field of neurodegenerative diseases.

## Figures and Tables

**Table 1:** Summary of Reviewed Soluble Epoxide Hydrolase Inhibitors.

**No.**	Drugs Used in Alzheimer’s Disease Studies	Mechanism of Action	Alzheimer’s Disease Research Findings	Experimental In Vivo Models Used in Alzheimer’s Disease Studies	Other Explored Neurological/Psychiatric Conditions	References
1.	TPPU	Potent sEH inhibitor with p38 blocking activity	The reduction of Aβ plaques, hyperphosphorylated tau, neuroinflammation, astrogliosis, microglial activation, and oxidative stress. Improved cerebral blood flow, neuroprotection, memory, and cognitive function.	Rodents: Familial Alzheimer’s Disease (5xFAD) mice Senescence-Accelerated Mouse-Prone 8 (SAMP8) C57BL/6 mice CD1 mice Sprague-Dawley rats TgF344-AD rats PS19 Tau P301S transgenic mice Doses: 1–5 mg/kg/day Route of Administration: Oral, intraperitoneal injections Duration: 2–20 weeks Insects: Aβ42 transgenic Drosophila.	Stroke Parkinson’s disease Depression Epilepsy Neurodevelopmental disorders	[13,61,69,70,77,78,79,81,85,92,93,94,95,96,97,98,110,111,112,113,114,115,116,117,118,119,120,121,122,123,124,125,126,127,128,129]
2.	TPPU-6-chlorotacrine	A hybrid of TPPU and 6-chlorotacrine (huperine Y), an acetylcholine esterase inhibitor	The reduction of Aβ plaques, hyperphosphorylated tau, and neuroinflammation. Improved memory and cognitive function.	Mice (SAMP8) Dose: 2 mg/kg/day Route of Administration: Oral Duration: 4 weeks	-	[104]
3.	UB-SCG-51	Potent and selective sEH inhibitor derived from Benzohomoadamantane	The reduction of Aβ plaques, hyperphosphorylated tau, neuroinflammation, astrogliosis, microglial activation, and oxidative stress. Improved neuroprotection, memory, and cognitive function.	5XFAD mice. Dose: 5 mg/kg/day Route of Administration: Oral Duration: 4 weeks	-	[100]
4.	UB-SCG-55	Safe and potent sEH inhibitor derived from Benzohomoadamantane	The reduction of neuroinflammation and microglial inflammation.	Experiments were performed in vitro: (Primary microglial and BV2 cells (murine)).	-	[77]
5.	UB-SCG-74	Selective, potent, and safe sEH inhibitor derived from Benzohomoadamantane. Has good oral absorption and BBB permeability.	Improved memory, cognitive, and synaptic function.	5XFAD mice. Doses: 0.5, 1.5, and 3 mg/kg/day Route of Administration: Oral Duration: 4 weeks	-	[101]
6.	UB-BJ02	Novel soluble epoxide hydrolase inhibitor	The reduction of Aβ plaques, neuroinflammation, astrogliosis, microglial activation. Improved memory and cognitive function. Improved mitochondrial function and gut microbiota diversity	5XFAD mice Dose: 5 mg/kg/day Route of Administration: Oral Duration: 4 weeks	-	[102]
7.	AS-2586114 and UB-EV-52	Newer inhibitors with BBB crossing ability	The reduction of Aβ plaques, hyperphosphorylated tau, neuroinflammation, and oxidative stress. Improved memory and cognitive function.	SAMP8 and 5XFAD mice Doses: AS-2586114: 7.5 mg/kg/day UB-EV-52: 5 mg/kg/day Route of Administration: Oral Duration: 4 weeks	Antipsychotic activity (AS-2586114) Cognitive decline in Niemann-pick disease (UB-EV-52)	[13,130,131]
8.	UB23 and UB28	Novel sEH inhibitors	The reduction of neuroinflammation. Improved neuroprotection.	Experiments were performed in vitro: SH-SY5Y cells Murine microglial cells (BV2).	-	[105]
**Other Drugs**						
9.	AUDA	sEH inhibitor with anti-inflammatory effects	Protective against stroke through inhibition of EETs synthesis, microglial M2 polarization. Has anti-inflammatory, antioxidant, and neuroprotective properties.	C57Bl/6 mice. Wistar Kyoto rats. Dose: 10–20 mg/kg/day Route of Administration: Intraperitoneal Duration:1–2 days	Intracerebral hemorrhage Brain and spinal cord injury Postpartum depression Epilepsy	[57,58,126,132,133,134,135,136]
10.	Kurarinone	A natural flavonoid derived from *Sophora flavescens*, with a non-competitive sEH inhibitory function.	Showed behavioural alleviation in Parkinson’s disease-, reduced neurotoxicity and neuroinflammation.	C57BL/6 mice Dose: 5–10–20 mg/kg/day Route of Administration: Oral Duration: 12–19 days	Insomnia Neurodegeneration Cerebral ischemia. Cerebral hemorrhage	[137,138,139,140,141,142,143]
11.	t-AUCB	Potent sEH inhibitor	Protective against DM-related cognitive dysfunction, through antioxidation and neuroprotection.	db/db mice Dose: 2 mg/L in drinking water (0.6–0.8 μg/kg/day) Route of Administration: Oral Duration: 4 weeks	Glioblastoma Scrapie Stroke Metabolic neurological complications	[82,144,145,146,147,148,149,150,151,152,153,154,155,156,157,158,159,160,161,162]
